# Characteristics of Hospitalized Elderly Patients with Severe Pneumonia Due to SARS-CoV-2, Vaccinated Against COVID-19

**DOI:** 10.3390/life15060879

**Published:** 2025-05-29

**Authors:** Jakub Kisiel, Michał Chojnicki, Arleta Kowala-Piaskowska, Katarzyna Wieczorowska-Tobis, Sławomir Tobis, Urszula Religioni, Piotr Merks, Agnieszka Neumann-Podczaska

**Affiliations:** 1 Multispecialist Municipal Hospital, J. Struś Memorial Hospital, Szwajcarska 3, 61-285 Poznan, Poland; jakub.kisiel@wp.pl; 2 Department of Medical Biology, Poznan University of Medical Sciences, Rokietnicka 10, 60-806 Poznan, Poland; 3 Department and Clinic of Infectious Diseases, Hepatology and Acquired Immunodeficiencies, Poznan University of Medical Sciences, Szwajcarska 3, 61-285 Poznan, Poland; 4 Department of Palliative Medicine, Poznan University of Medical Sciences, 61-285 Poznan, Polandar-n@wp.pl (A.N.-P.); 5 Department of Occupational Therapy, Poznan University of Medical Sciences, 60-781 Poznan, Poland; 6 School of Public Health, Centre of Postgraduate Medical Education of Warsaw, 01-826 Warsaw, Poland; 7 Department of Pharmacology and Clinical Pharmacology, Faculty of Medicine, Collegium Medicum, Cardinal Stefan Wyszyński University, 01-815 Warsaw, Poland; 8 Senior Institute, University of Economics and Humans Sciences in Warsaw, 01-043 Warsaw, Poland

**Keywords:** COVID-19 vaccination, COVID-19, SARS-CoV-2, interstitial pneumonia, older adults, comorbidities, multimorbidity, hospitalization, mortality

## Abstract

The introduction of COVID-19 vaccinations has significantly altered the course of the pandemic by markedly reducing the number of severe infection cases, hospitalizations, and deaths due to COVID-19. Elderly individuals constitute a particularly vulnerable group at risk of severe disease progression, which is often related to decreased immune system effectiveness and comorbidities. Severe infection outcomes in vaccinated individuals, though substantially rarer than in the unimmunized population, can still lead to death due to underlying health conditions. This analysis aims to describe the population of elderly individuals who, despite being vaccinated, died from interstitial pneumonia complicating SARS-CoV-2 infection. Data on the infection course and co-existing diseases were obtained from the database of the Józef Struś Multispecialty City Hospital in Poznań, which was converted into a dedicated facility during the pandemic. The inclusion criteria for the analysis were being over 60 years of age on the day of hospital admission, confirmed pneumonia in radiological examination, COVID-19 infection confirmed by PCR test, and an adverse disease course resulting in death. Patients admitted to the hospital from 1 June 2021 to 31 December 2021 were analyzed. Out of all hospitalizations, only 18 individuals met the inclusion criteria. Given the small number of patients, the authors employed descriptive methods to illustrate the clinical states of the individual patients, presenting SARS-CoV-2 infection in the context of co-existing diseases that significantly affect prognosis. The qualitative analysis employed highlights the complex and multidimensional courses of severely ill COVID-19 patients more emphatically.

## 1. Introduction

The World Health Organization (WHO) declared the end of the COVID-19 pandemic on 5 May 2023 [1]. This global health crisis, which had been ongoing since early 2020, dominated public discourse and political actions worldwide, causing unprecedented societal changes. The health risks associated with the spread of SARS-CoV-2 and healthcare system responses were the subject of intensive research and discussion. Systems were forced to adapt, emphasizing various preventive measures from mass vaccination to social isolation policies [2].

From the beginning of the pandemic, older individuals were identified as being at higher risk of severe COVID-19. Observations from 2020 indicated that people over 65 years of age accounted for 80% of hospitalizations, with a mortality risk 23 times higher than that of younger individuals. It was noted that age as an independent risk factor was not entirely explained by coexisting diseases such as cardiovascular disease, diabetes, or obesity [3]. These findings quickly translated into concrete actions at both political and practical levels, leading to initiatives aimed at protecting this population.

The most crucial measure introduced for prevention was active immunization. Elderly individuals were prioritized for vaccination access, resulting in a significant reduction in severe cases and deaths. Mass vaccination efforts began in December 2020, initially with three vaccines: Pfizer–BioNTech (BNT162b2), Moderna (mRNA-1273), and AstraZeneca–Oxford (AZD1222) [4,5]. In Poland, all three vaccines were used in the national vaccination program, which started in January 2021 and prioritized the elderly before expanding to other groups.

Israel was one of the first countries to implement mass vaccination programs, showcasing the effectiveness of immunization [6]. Data revealed that vaccination significantly reduced the risk of severe illness and death from COVID-19 [6]. However, it is important to note that vaccines did not provide absolute protection against infection. Even with a high percentage of the population vaccinated, the transmission of SARS-CoV-2 continued, particularly with the emergence of new variants like Delta. This situation emphasized the complexity of pandemic dynamics and the ongoing necessity for additional public health measures.

Recent observational studies highlight the life-saving benefits of vaccination for older adults. For example, data from Israel showed that in individuals aged 60 and older, full vaccination reduced COVID-19-related mortality by nearly 10 to 20 times compared with unvaccinated individuals [6]. Similarly, large-scale studies in the United States reported that fully vaccinated seniors experienced approximately an 85% reduction in hospitalizations and up to a 90% decrease in mortality [7]. These significant reductions emphasize vaccines’ critical role in protecting those most at risk. Before vaccinations were available, older adults faced extraordinary vulnerability, with mortality rates up to 23 times higher than those in younger age groups [8]. Even a modest increase in vaccination coverage can lead to substantial public health benefits.

Booster doses are crucial for maintaining high levels of protection, especially as immunity wanes and new variants emerge. Observational data consistently demonstrate that additional booster doses restore vaccine effectiveness, ensuring that older individuals have significantly lower risks of severe outcomes and death. These findings emphasize the need for ongoing and targeted vaccination campaigns for the elderly [8].

## 2. Study Aim

Despite the high effectiveness of vaccination in preventing severe SARS-CoV-2 infections, some fully vaccinated individuals still succumbed to the disease. These fatalities were often associated with pre-existing health conditions, including cardiovascular diseases that lead to heart attacks or strokes. In some cases, deaths occurred directly due to pneumonia caused by SARS-CoV-2 [6].

This study aims to retrospectively analyze the infection courses and coexisting diseases in individuals over 60 years of age whose deaths were attributed to pneumonia following SARS-CoV-2 infection.

A key reason for expanding this research is to directly address the growing anti-vaccination movements that challenge the effectiveness of immunization. Conducting comprehensive analyses of cases in which vaccination appears to have failed is vital in countering the narratives put forth by organizations such as the National Vaccine Information Center, Children’s Health Defense, and Stop Mandatory Vaccination, which operate on a global scale.

By systematically examining these cases, researchers can generate credible data that not only affirm the overall effectiveness of vaccines but also provides compelling evidence to refute misleading claims. Evidence-based approaches are essential for effective public health communication and for fostering informed discussions with skeptics across various regions.

## 3. Material and Methods

A retrospective study was designed to analyze the hospitalization data obtained from the electronic system of the Multispecialty Municipal Hospital named after Józef Struś in Poznań. During the pandemic, this facility was transformed into a dedicated COVID-19 hospital specializing in the treatment of patients infected with SARS-CoV-2. Due to the complex health conditions of the patients, this study was extended to include case descriptions, characterizing patients who died during hospitalization.

The analysis included all individuals hospitalized between 1 June 2021 and 31 December 2021 who were at least 60 years old at the time of hospital admission. Admission to the hospital required a confirmed COVID-19 diagnosis by RT-PCR. From the group of hospitalized patients, those with radiologically confirmed pneumonia (via computed tomography or chest X-Ray) were included in this study.

This study analyzed data on the pre-hospitalization period, the hospital laboratory test results, and patient symptoms. Symptoms assessed included dyspnea, cough, nasopharyngeal congestion, weakness, myalgia, cognitive impairment, and fever. The routinely tested laboratory parameters included, among others, the presence of specific blood smear components, lymphocyte count, interleukin-6 levels, creatinine and urea concentrations, vitamin D3 levels, aminotransferase activity, C-reactive protein levels, procalcitonin levels, and coagulation parameters, including D-dimer levels. These laboratory results were obtained both at hospital admission and at the peak severity of the disease.

## 4. Results

### 4.1. Description of the Study Group

During the study period, 305 individuals aged 60 years and older were hospitalized. Radiological signs of pneumonia were observed in 235 of them, accounting for 77% of this group. More than half of the patients (52%) had not received any dose of the COVID-19 vaccine. Every fifth patient (20.4%) was fully vaccinated, while the remaining individuals had either received a single dose or had been immunized more than six months prior to hospitalization. Among fully vaccinated patients, 18 died during hospitalization, representing 7.7% of those diagnosed with pneumonia (Table 1).

The analyzed group included more women than men, with the youngest patient being a 63-year-old woman and the oldest a 98-year-old female patient.

Among the fully vaccinated patients who died, the Charlson Comorbidity Index (CCI) ranged from 1 to 7, with an average value of 4.22 (SD = 1.83) and a median of 4.5. These figures suggest that the population was heavily burdened with chronic illnesses and high levels of multimorbidity. However, it is important to note that the CCI does not encompass all factors that may have contributed to mortality in this study. For example, it does not take into account iatrogenic immunosuppression or other clinical nuances that could have influenced the outcomes.

### 4.2. Length of Hospitalization

The duration between hospital admission and death varied significantly, ranging from 1 to 49 days, with an average of 15.82 days. The standard deviation (SD) was 13.69, the median was 10 days, and the mode was 7 days. The high SD values indicate substantial differences in the length of hospital stays, reflecting variations in the patients’ initial health conditions and comorbidities (Table 2).

### 4.3. Condition at Admission

Most patients (65%) were admitted from home, while 10% came from nursing homes or long-term care facilities (DPS/ZOL), 20% were transferred from another hospital, and 5% from a palliative care unit.

The most frequently diagnosed chronic condition at admission was hypertension, which was present in 15 patients (79%). Additionally, six patients (32%) had diabetes, 14 patients (74%) had cardiovascular diseases other than hypertension, and five patients (26%) had kidney disease. One patient was diagnosed with advanced liver cirrhosis due to HBV infection.

At the time of hospital admission, patients presented the following symptoms:

- Fever in two patients (11%),
- Low-grade fever in 10 patients (53%),
- Cough in 11 patients (58%),
- Dyspnea in 14 patients (74%),
- Muscle pain in two patients (11%),
- No cases of nasal congestion, taste, or smell disorders,
- Weakness in 15 patients (79%),
- Cognitive impairment at admission in eight patients (42%),
- Chronic disease exacerbation in 17 patients (89%).

Based on nursing and physician observations, as well as hospital records, each patient’s’ functional status at admission was assessed using the ECOG (Eastern Cooperative Oncology Group) performance scale. Seven patients were classified at ECOG level 2, eight at level 3, and four at level 4 (requiring constant, around-the-clock care and being bedridden). No patient was assessed as having normal activity or requiring only minimal support (ECOG levels 0 and 1).

At the time of admission, the following oxygen saturation levels were recorded:

- Below 70% in one patient,
- Between 71–80% in two patients,
- Between 81–90% in four patients,
- Above 90% in the remaining 11 patients.

### 4.4. Biochemical Test Results

Significantly elevated C-reactive protein (CRP) levels were observed in all patients, serving as a criterion for pneumonia diagnosis. Lymphopenia, characteristic of the early phases of viral infection, was recorded in six patients (33%). Interleukin-6 levels were elevated in 11 patients (61%), indicating ongoing infection. Similarly, procalcitonin, which correlates with bacterial infections, was elevated in 12 patients (66.6%). Vitamin D3 levels were not measured in all patients; however, among those tested, 11 out of 14 had significantly low levels, indicating widespread deficiency in this population.

### 4.5. Neurological Chronic Diseases

In the analyzed cohort, six patients had documented histories of transient ischemic attack (TIA) or stroke, and one patient presented with cerebrovascular disorders upon hospital admission. During their hospitalizations, cognitive impairment was identified in all patients (100%). Neurological abnormalities were observed either as primary disorders—such as frontotemporal dementia, Parkinson’s disease, and cerebral palsy—or as secondary complications resulting from chronic medical conditions such as advanced heart failure, severe generalized atherosclerosis, or poorly controlled type 2 diabetes mellitus.

Several specific neurological conditions were noted among the patients. One was a 74-year-old man who had a history of ischemic stroke, advanced dilated cardiomyopathy with heart failure (HFrEF), insulin-treated diabetes mellitus, and anemia due to vitamin B12 deficiency. Another patient, a 70-year-old woman, had frontotemporal dementia, a history of surgery for an ependymoma in 2009, and a previous ischemic stroke that resulted in persistent left-sided hemiparesis. Additionally, a 62-year-old woman had lifelong cerebral palsy, which was complicated by morbid obesity, severe generalized atherosclerosis, and multiple cardiovascular comorbidities.

Older patients, particularly those between 88 and 97 years of age, exhibited neurological symptoms mainly due to chronic cerebral hypoperfusion, which is linked to extensive cardiovascular diseases such as advanced peripheral artery disease and persistent atrial fibrillation. For these individuals, cognitive impairment was likely exacerbated by factors like chronic immobility, chronic anemia, and nutritional deficiencies.

In summary, the neurological assessment of this patient group indicated a high prevalence of cerebrovascular events, dementia syndromes, and chronic neurological conditions, all of which significantly impacted their prognoses. In the context of COVID-19, these neurological comorbidities further elevated the risk of severe disease progression and mortality in this particularly vulnerable population.

### 4.6. Chronic Vascular and Cardiac Diseases

All patients in the study group were diagnosed with cardiovascular diseases during hospitalization. Severe forms of these diseases were identified in nine patients, including the following:

- Advanced heart failure classified as NYHA stage 2 or 3,
- Severe dilated cardiomyopathy,
- Mitral valve and pulmonary trunk stenosis,
- Complete atrioventricular block (third-degree heart block).

Cardiovascular conditions have been identified as a major factor contributing to mortality. A thorough review of patient records revealed that many individuals suffered from severe cardiac diseases, which likely played a significant role in their fatal outcomes.

For instance, a 64-year-old patient (Patient 1) experienced a ST-segment elevation myocardial infarction (STEMI) during a coronary angiography procedure, which was accompanied by cardiac arrest. This incident suggests the presence of underlying ischemic heart disease and heart failure.

A 74-year-old patient (Patient 2) experienced multiple documented cardiac events, including a non-ST-elevation myocardial infarction (NSTEMI), a prior myocardial infarction, and episodes of ventricular fibrillation that resulted in cardiac arrest. This patient also had a history of dilated cardiomyopathy and had undergone pacemaker implantation, indicating the advanced nature of his cardiac condition.

Additional cases within the cohort revealed significant cardiovascular issues. Patients aged 74, 80, 89, and 97 presented with chronic heart failure, ischemic heart disease, and arrhythmias such as atrial fibrillation, along with valvular abnormalities including mitral stenosis and tricuspid regurgitation. Notably, an 88-year-old patient died due to resistant bradyasystole, indicating a fatal cardiac event.

Collectively, these findings suggest that a substantial portion of the observed deaths can be attributed at least in part to severe cardiovascular conditions. Overall, the high prevalence and severity of these cardiac disorders emphasize the fact that, in addition to respiratory complications from COVID-19 pneumonia, underlying cardiovascular diseases likely contributed significantly to the mortality observed in this high-risk population. This observation is especially important as it demonstrates that, despite the overall effectiveness of COVID-19 vaccination in reducing severe disease and death, elderly patients with extensive cardiac comorbidities remain particularly vulnerable. Consequently, these findings highlight the necessity for comprehensive cardiovascular evaluation and targeted management strategies in the care of older COVID-19 patients, even among those who are fully vaccinated.

### 4.7. Other Chronic Conditions

Diabetes was diagnosed in five patients (28%). Since the early months of the pandemic, diabetes has been identified as a factor contributing to worse COVID-19 outcomes. It also exacerbates atherosclerosis, increasing the risk of cardiovascular events.

Thyroid disorders (both hypo- and hyperthyroidism) requiring pharmacological treatment were identified in four patients. One patient had gout that required allopurinol treatment. Rheumatic diseases were also present in some patients and could have influenced the course of infection. The prevalence of chronic diseases is presented in Table 3.

### 4.8. Radiological Examinations

Pneumonia diagnosis was based on radiological findings, including chest X-Rays or computed tomography (CT) scans, mostly without contrast. In some patients, the radiological reports also indicated persistent lung changes from previous infections, such as fibrosis.

### 4.9. Course of Hospitalization

Respiratory failure requiring mechanical ventilation occurred in 12 patients (66.6%). All patients experienced consciousness disorders during hospitalization.

Antiviral treatment with remdesivir, tocilizumab, or baricitinib was administered to six patients. Additionally, four patients received steroid therapy. All patients were treated with antibiotics.

### 4.10. Patient Descriptions

Table 4 provides detailed information on individual patients. The “Remarks” section contains specific details regarding hospitalization. Some data that could potentially identify patients have been removed.

## 5. Discussion

The effectiveness of COVID-19 vaccination has been clearly demonstrated in numerous retrospective studies. A meta-analysis conducted by a Chinese research team, encompassing 51 studies, confirmed the high real-world effectiveness of COVID-19 vaccines. According to the analysis, vaccination reduced SARS-CoV-2 infections by 89.1%, hospital admissions by 97.2%, ICU admissions by 97.4%, and mortality by 99.0%. Although vaccine effectiveness varied across age groups, it remained consistently high in all of them [8]. In the present study, the authors conducted a retrospective analysis of severe COVID-19 cases that resulted in the deaths of previously vaccinated individuals, where SARS-CoV-2 infection was recorded as the cause of death. The analysis identified additional critical comorbidities that significantly contributed to mortality. In most cases, these comorbidities were not directly attributable to SARS-CoV-2 infection. Primary causes of death in this group included events such as decompensated liver cirrhosis, heart failure, massive arterial bleeding following the removal of a Sheldon catheter, and multiple strokes leading to dementia syndrome. Furthermore, this study emphasizes that this patient population is particularly vulnerable to adverse outcomes of hospitalization, regardless of infection status. The second major cause of death identified was severe COVID-19 superimposed on pre-existing chronic conditions. Notable examples include morbid obesity (BMI > 40 kg/m^2^), pulmonary fibrosis, and complications acquired during hospitalization, such as Escherichia coli-induced sepsis, septicemia, and Clostridium difficile-associated diarrhea in immunosuppressed individuals. Other critical events included refractory bradyasystole and recurrent cardiac arrests in patients with significant cardiovascular disease. Although not directly linked to SARS-CoV-2 infection, these conditions played a substantial role in unfavorable clinical outcomes. The authors deliberately chose not to include a control group in the analysis, citing significant variability in functional status among elderly individuals, even within the same age group. Health conditions and baseline functional capacities differ markedly in this population, making age- and sex-matched comparisons potentially misleading. Such a comparison could inadvertently highlight the disproportionate burden of comorbidities among patients with unfavorable COVID-19 outcomes rather than elucidating meaningful differences. Consequently, this study prioritized a comprehensive characterization of the investigated cohort to provide clinically relevant insights. Fatal breakthrough infections predominantly occurred in multimorbid elderly patients, suggesting that host frailty—rather than vaccine failure—is the primary driver of post-vaccination mortality. These findings underscore the importance of prioritizing booster doses, ensuring timely administration of antiviral therapies, and maintaining rigorous management of chronic conditions within this vulnerable population.

Clear communication of this distinction is essential to counter anti-vaccine narratives. Emphasizing that vaccines continue to demonstrate high effectiveness at the population level, even as severely comorbid individuals remain at risk for adverse outcomes, can help preserve public trust in vaccination programs.

A key limitation of this study is the absence of comprehensive data on post-vaccination serological status. During hospitalization, antibody levels against the SARS-CoV-2 spike protein—an essential viral component and the principal target of most vaccines—were not routinely assessed. This lack of data limits insight into the immunological statuses of patients at the times of infection. Moreover, previous research indicates that individuals with compromised immune systems—such as transplant recipients, patients with autoimmune diseases receiving immunosuppressive therapy, and older adults—may exhibit diminished immune responses to vaccination [9,10]. This reduced immunogenicity can significantly affect both the course and treatment efficacy of SARS-CoV-2 infection in these groups, highlighting the need for more precise monitoring and tailored therapeutic and preventive strategies in pandemic management.

Another limitation of this study is the relatively small cohort size, comprising only 18 vaccinated patients who died from pneumonia. This low number likely reflects the high effectiveness of COVID-19 vaccines. Nevertheless, the small sample size severely limits statistical analysis and weakens the external validity of the findings, preventing robust conclusions regarding treatment efficacy or comorbidity patterns. This study focuses on a detailed clinical characterization of fatal cases, but generalizability is inherently restricted.

Importantly, this study lacks a formal comparison group. While the rationale for this design choice is explained, the absence of a control cohort limits the interpretive depth. Even a basic descriptive comparison, such as median Charlson Comorbidity Index (CCI) scores among survivors, could have strengthened the contextual relevance of the presented findings.

As a retrospective analysis, this study is constrained by the availability and completeness of data in electronic medical records. Frailty was not routinely assessed using standardized scales, treatment information was partially documented, and antibody testing was not systematically performed. These factors further limit the granularity of the analysis and may introduce bias in the interpretation of clinical outcomes.

Additionally, while the assumption of Delta variant predominance during the study period is plausible and supported by national surveillance data, specific variant confirmation was not conducted. This weakens the strength of the conclusions, particularly if variant-specific responses become a standard focus in future research.

Lastly, this study was conducted in a single Polish hospital, which may limit the global applicability of the findings due to cultural, systemic, and organizational differences in healthcare delivery. These contextual factors, together with the retrospective design and small sample size, underscore the need for cautious interpretation and highlight the importance of further multicenter prospective studies.

## 6. Conclusions

1. Fatal outcomes from COVID-19 in older, fully vaccinated patients are mainly associated with severe and advanced comorbidities.
2. Severe pneumonia in this population often results from the exacerbation of chronic conditions, posing significant challenges for hospital and intensive care management.
3. Comprehensive care strategies tailored to elderly patients with multimorbidity remain crucial, as vaccination alone does not fully eliminate the risk of severe SARS-CoV-2 infection.
4. Public health policies should consider timely administration of booster doses, routine frailty screening, and improved access to early treatment options for older adults.
5. Future research should focus on larger, multicenter prospective studies that incorporate detailed immunological assessments to clarify the interactions between vaccine-induced immunity, patient-specific factors, and clinical outcomes in this vulnerable group.

## Figures and Tables

**Table 1:** Patients’ data.

Parameter	Number of Patients
Hospitalized patients (aged ≥60 years)	305
With radiologically confirmed pneumonia	235
Fully vaccinated against COVID-19	62
Included in the analysis (met all criteria)	18

**Table 2:** Baseline characteristics of the 18 patients. The cohort consisted of 10 males (55.6%) and 8 females (44.4%).

Analyzed Characteristic	Mean	Median	Standard Deviation (SD)	Minimum	Maximum	Interquartile Range (IQR)	Mode (s)
Length of Stay (days)	≈13.28	11.5	≈7.50	1	27	12	12
Patient Age (years)	≈76.5	75	≈9.88	62	97	15	69

**Table 3:** Prevalence of chronic disease categories in the patient cohort (N = 18).

Disease Category	Number of Patients (out of 18)
Cardiovascular diseases	13
Hypertension	13
Neurological disorders	9
Diabetes mellitus	6
Other metabolic/endocrine disorders (excluding Diabetes)	6
Gastrointestinal and hepatic diseases	5
Renal diseases	4
Rheumatic and musculoskeletal diseases	4
Respiratory diseases	3
Anemia	3
Non-cardiac/non-neurological vascular diseases	3
Neoplastic diseases	2
Patients with no listed chronic diseases	1

**Table 4:** Detailed information on individual patients.

Gender	Age	Comment	Chronic Diseases
M	64	On the 4th day of hospitalization, the patient suffered a severe heart attack, and during coronary angiography, sudden cardiac arrest occurred.	Hypertension, status: post-acute coronary syndrome
M	74	Two months before hospitalization, the patient was diagnosed with SARS-CoV-2 infection.	Bladder tumor, sudden cardiac arrest (ventricular fibrillation, 2021), dilated cardiomyopathy, status: post-pacemaker implantation, status post-myocardial infarction, status: post-stroke, type 2 diabetes, vitamin B12 deficiency anemia
F	84	During hospitalization, the patient was diagnosed with sepsis caused by E. coli ESBL+ strain, clostridium difficile infection, and diverticulitis; the patient underwent dialysis therapy.	End-stage renal disease, heart failure NYHA II, status: post-pacemaker implantation due to third-degree atrioventricular block, type 2 diabetes, diverticular disease
M	88	The cause of the patient’s death was refractory bradyasystolia.	Hearing loss
M	81	Multiple hemorrhages, including arterial bleeding after Sheldon catheter removal and respiratory tract bleeding.	Chronic kidney disease (dialysis patient), tricuspid valve regurgitation, coronary artery disease, hypertension, anemia, hypercholesterolemia, prostate hypertrophy
M	69	During a pulmonology consultation, pulmonary fibrosis was suspected.	Chronic kidney disease, hypertension
F	73		Hypothyroidism, type 2 diabetes, hypertension, arrhythmia
F	70		Frontotemporal dementia, hypertension, aortic valve regurgitation (grade II), status post-ependymoma surgery (2009), benzodiazepine addiction, ischemic stroke with left-sided paresis (2016)
M	74	During hospitalization, the patient also developed sepsis of streptococcus pneumoniae etiology and clostridium difficile-related diarrhea.	Heart failure, status: post two myocardial infarctions (last infarction in 2020), type 2 diabetes treated with insulin, chronic obstructive pulmonary disease (group D), hypertension, long-term smoking
M	76		History of strokes, left-sided hemiparesis and dysarthria, umbilical hernia, hypertension
M	67	Three sudden cardiac arrests, three resuscitations.	Hypertension, rheumatoid arthritis
F	62	Patient with cerebral palsy: one week before admission, they suffered a severe urinary tract infection requiring hospitalization.	Atherosclerosis, morbid obesity, coronary artery disease, supraventricular arrhythmias, acquired mitral valve disease (mild regurgitation), hypertension, type 2 diabetes, gout, chronic venous insufficiency of lower limbs, severe vitamin D deficiency, spinal degenerative disease, cerebral palsy, acute pancreatitis (October 2019), bilateral pneumonia with pleural empyema (2019)
M	80		Status post-myocardial infarction (2018), atrial fibrillation, hypertension, status: post-stroke, gallbladder stones, kidney failure, Parkinson’s disease, status: post-right hip and left knee endoprosthesis, degenerative joint disease
F	76	The patient was admitted due to esophageal variceal bleeding with coexisting respiratory failure.	HBV infection, decompensated liver cirrhosis (esophageal varices, hepatic encephalopathy), electrolyte disturbances (hyponatremia, hypokalemia), circulatory failure, vascular brain damage, hiatal hernia, type 2 diabetes, hypertension
M	84		Dementia syndrome, status: post-stroke, status: post-myocardial infarction, COPD
F	69		None
F	89		Hypertension, atrial fibrillation, gout, status: post-stroke (2017), deep vein thrombosis, mitral valve disease (moderate stenosis), coronary artery disease, lipid disorders
F	97		Anemia of chronic diseases, Chronic heart failure NYHA II, permanent atrial fibrillation, peripheral artery disease, status: post-lower limb amputation due to acute ischemia (2019), varicose vein disease of lower limbs, Stage 2 venous insufficiency, hypertension, hyperthyroidism treated with thiamazole, pressure ulcer

## Data Availability

All data are available from the corresponding author.
